# A New Insight into Coating’s Formation Mechanism Between TiO_2_ and Alendronate on Titanium Dental Implant

**DOI:** 10.3390/ma13143220

**Published:** 2020-07-20

**Authors:** Željka Petrović, Ankica Šarić, Ines Despotović, Jozefina Katić, Robert Peter, Mladen Petravić, Marin Petković

**Affiliations:** 1 Division of Materials Chemistry, Ruđer Bošković Institute, Bijenička cesta 54, 10002 Zagreb, Croatia; 2 Division of Materials Physics, Centre of Excellence for Advanced Materials and Sensing Device, Ruđer Bošković Institute, Bijenička cesta 54, 10002 Zagreb, Croatia; 3 Division of Physical Chemistry, Ruđer Bošković Institute, Bijenička cesta 54, 10002 Zagreb, Croatia; Ines.Despotovic@irb.hr; 4 Department of Electrochemistry, Faculty of Chemical Engineering and Technology, University of Zagreb, Marulićev trg 19, 10000 Zagreb, Croatia; jkatic@fkit.hr; 5 Department of Physics and Center for Micro- and Nanosciences and Technologies, University of Rijeka, R. Matejcic 2, 51000 Rijeka, Croatia; rpeter@phy.uniri.hr (R.P.); mpetravic@phy.uniri.hr (M.P.); 6 Adentro dental studio, Petrova ul. 67, 10000 Zagreb, Croatia; info@adentro.hr

**Keywords:** titanium dental implant, alendronate sodium, surface coating, DFT, XPS, EIS

## Abstract

Organophosphorus compounds, like bisphosphonates, drugs for treatment and prevention of bone diseases, have been successfully applied in recent years as bioactive and osseoinductive coatings on dental implants. An integrated experimental-theoretical approach was utilized in this study to clarify the mechanism of bisphosphonate-based coating formation on dental implant surfaces. Experimental validation of the alendronate coating formation on the titanium dental implant surface was carried out by X-ray photoelectron spectroscopy and contact angle measurements. Detailed theoretical simulations of all probable molecular implant surface/alendronate interactions were performed employing quantum chemical calculations at the density functional theory level. The calculated Gibbs free energies of (TiO_2_)_10_–alendronate interaction indicate a more spontaneous exergonic process when alendronate molecules interact directly with the titanium surface via two strong bonds, Ti–N and Ti–O, through simultaneous participation common to both phosphonate and amine branches. Additionally, the stability of the alendronate-modified implant during 7 day-immersion in a simulated saliva solution has been investigated by using electrochemical impedance spectroscopy. The alendronate coating was stable during immersion in the artificial saliva solution and acted as an additional barrier on the implant with overall resistivity, *R* ~ 5.9 MΩ cm^2^.

## 1. Introduction

State-of-the-art investigations of orthopedic and dental implants are focused on bone-inspired surface modification [1,2,3,4]. The success of implantation, which is complex and time-consuming process, depends greatly on osseointegration [5,6]. This is a process of creation of structural and functional connections between the implant and surrounding bone tissue; therefore, it is of great interest to functionalize the implant surface to induce, promote, and accelerate the osseointegration. At the same time, the modified implant surface must satisfy several other important characteristics, such as non-toxicity, non-allergy, possess adequate mechanical and anti-corrosion protection, etc., since implants are foreign bodies for the human organism. Hence, it is very challenging to find molecules/substances that will meet all requirements.

One of strategies is to implant surface modification by inorganic coatings, like calcium phosphates (CaP) or hydroxyapatite (HA), that represent inorganic phase of the natural bone [1,2,4,7,8,9,10,11,12]. HA-based coatings on the titanium implants are responsible for osteoconductivity, bioactivity, and stability of the bone/implant connection. To produce CaP-based coatings more similar to natural bones, which normally contain different inorganic ions, ions, like magnesium, zinc, or titanium, are added to the CaP phase. So, magnesium ions regulate inflammatory activity [13], zinc ions stimulate new bone formation and antimicrobial and anti-inflammatory activities [14,15], and titanium ions increase the proliferation of osteoblasts [16]. Bioceramic coatings, like sphene-based (CaTiSiO_5_) coatings, can promote early cell/implant surface interactions and osteoblast proliferation and differentiation due to Ca and Si dissolution from the sphene coating [17,18]. In addition, bioactive glasses are used as bioactive coatings for bone scaffolds [19] and for porous titanium implants [20]. Coatings based on the organic bone phase are also used for improving implant/bone responses [1,2,4]. For bone healing, coatings with different extracellular matrix (ECM) proteins (collagen, fibronectin, fibrinogen) are applied [4,21], and a relatively new approach is a combination of inorganic and organic phases of bones, like HA and collagen, which enhances adhesion, proliferation, and differentiation of Mesenchymal Stem Cells (MSCs) [22]. In order to shorten time required for implant fixation, as well as to enhance bone regeneration around implant, recent studies have focused on biomimetic coatings by using biomolecules, such as platelet-derived growth factor (PDGF) [23] or heparin/dopamine molecules [24].

Recently, numerous in vitro, in vivo, and clinical studies about coatings that mitigate implant response, i.e., coatings induced by bone’s immune system, have been reported [1,2,3,4]. Since the implantation procedure destroys part of the bones and surrounding tissues and often evokes an inflammatory reaction, thus limiting the implant response, researchers are focused on improving the osseonintegration of implants by using pharmaceutical compounds, like bisphosphonates, as bioactive and osteoinductive coatings [1,2,4,25,26,27,28,29]. The bisphosphonates (BP) class of synthetic drugs is frequently used for the treatment of bone diseases; osteoporosis, bone cancer, osteopenia, and Paget’s disease [2,7,30,31,32]. The BP’s pharmacological effect is related to their strong affinity for binding to the bone mineral phase and biochemical effect on bone cell, dominantly osteoclasts. They induce apoptosis of osteoclasts and thus favor bone formation over bone resorption [2,25,26,30,31].

Among BPs, alendronate, zolendronate, and pamidronate are the most tested molecules as coatings for implants [7,29,33,34,35,36,37]. It has been shown that alendronate-modified titanium acts as a bioactive implant that enhances simultaneously osteoblast differentiation and inhibits osteoclast differentiation [30,31], as well as enhances calcium deposition in the first ten days after implantation [35]. Functionalization of HA-coated titanium with BPs stabilizes implants in rats [38]. Compared to HA-modified surfaces that improve the binding of the implant to the bone, BP-modified surfaces favor new bone formation around implant [39].

Although the results of numerous studies show a positive osseoinductive influence induced by modification of implant surfaces with biphosphonate molecules, there is a lack of data crucial for fundamental understanding of the coating formation. Hence, the density functional theory (DFT calculations) corroborated by experimental findings of X-ray photoelectron spectroscopy (XPS) and contact angle (CA) measurement was applied to predict the mechanism of alendronate coating formation on the titanium-based dental implant. Since bioactive coatings, besides good osseoinductivity, have to possess certain characteristics necessary for their long-term life in the human body, anti-corrosion protection of the alendronate-modified implant during exposure to the artificial saliva was studied by electrochemical impedance spectroscopy (EIS).

## 2. Materials and Methods

### 2.1. Materials, Chemicals, and Solutions

**Figure 1:**
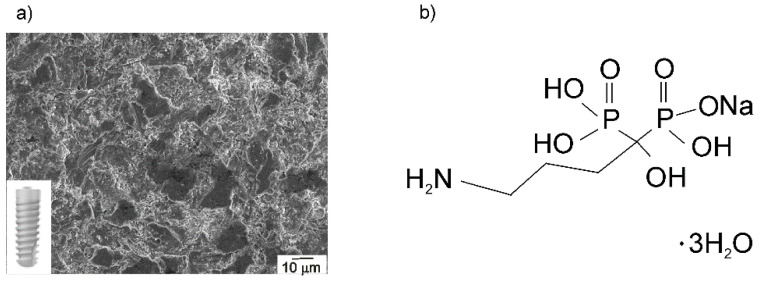
(**a**) The morphology of the Ankylos^®^ implant surface recorded at 500× magnification. The inset: the photography of the used dental implant C/X A11; (**b**) the structure of the sodium alendronate trihydrate.

The dental implant (Ankylos^®^ C/X A11; length: 11 mm, diameter: 3.5 mm, Dentsply Friadent^®^ GmbH, Mannheim, Germany), made of titanium grade 2 [40], was used as a substrate, shown in Figure 1a. The implant chemical composition can be found in Table 1, and its morphology, investigated by a field emission scanning electron microscopy, is visible in Figure 1a. The observed microstructure is a result of grit-blasting and high-temperature etching, and it is known as Friadent^®^ plus surface according to the producer’s data [40]. Before surface modification and each measurement, no treatments other than degreasing (see Section 2.2) were used to prepare the implant surface.

The aqueous solution of sodium alendronate trihydrate (Merck Sharp & Dohme, Kenilworth, NJ, USA, Figure 1b), prepared with Milli-Q^®^ water (Millipore, Merck, Darmstadt, Germany) in a concentration of 10 mmol dm^−3^, served as a solution for modification.

### 2.2. Alendronate Coating Formation on the Implant Surface

The surface of as-received implant was degreased with acetone (p.a., Gram-Mol, Zagreb, Croatia) and absolute ethanol (p.a., Gram-Mol, Zagreb, Croatia), and rinsed with Milli-Q^®^ water. The implant thus prepared was then immersed in a 10 mmol dm^−3^ alendronate solution (pH = 4) at 22 ± 2 °C for 24 h. After time elapsed, the modified implant was thermally treated at 70 °C for 5 h to enhance coating’s adhesion to the implant surface. This thermal step converts an adsorbed intermediate by an acid-base condensation reaction to chemically bonded coating to the implant surface [42,43,44]. Milli-Q^®^ water and absolute ethanol were used to rinse the modified sample, which was then dried in a stream of nitrogen (99.999%, Messer, Bad Soden, Germany).

### 2.3. Characterization of Implant Samples

The morphology was investigated by a field emission scanning electron microscope (JSM-7000F, Jeol Ltd., Tokyo, Japan) at 10 kV.

The wetting properties were examined by an OCA 20 contact angle system (Dataphysics Instruments GmbH, Filderstadt, Germany) at 22 ± 2 °C. Values are the average of three measurements of 1 µL Milli-Q^®^ water drop taken after a 10 s-stabilization period.

The SPECS instrument, equipped with the Phoibos MCD 100 electron analyzer and the monochromatized X-ray source of the energy of 1486.74 eV (Al Kα line), was used for X-ray photoelectron spectroscopy measurements. The photoemission spectra around Ti 2*p*, O 1*s*, and C 1*s* core-levels were recorded with the pass energy of 10 eV, while the 20 eV pass energy was used for the measurements around N 1*s* and P 2*p* core-levels. Measurements were carried out at 10^−7^ Pa. Experimental curves were deconvoluted using the mixed Gaussian–Lorentzian functions with Shirley background subtraction using Unifit software (ver. 2017) [45]. The calibration of binding energy (BE) scale was done against the BE of C 1*s* peak, placed at 285.0 eV.

The method of electrochemical impedance spectroscopy (EIS) was applied to investigate the implant stability during exposure to a Fusayama artificial saliva solution (0.4 g dm^−3^ NaCl, 0.4 g dm^−3^ KCl, 0.6 g dm^−3^ CaCl_2_·2H_2_O, 0.58 g dm^−3^ Na_2_HPO_4_·2H_2_O, and 1 g dm^−3^ urea; pH 6.8 [46]) at the open circuit potential (*E*_OCP_). The implant sample served as a working electrode (an area of 0.98 cm^2^), an Ag|AgCl, 3.0 mol dm^−3^ KCl (*E* = 0.210 V vs. standard hydrogen electrode (SHE)) as a reference, and a platinum plate as a counter electrode. Measurements were performed between 10^4^ and 10^−3^ Hz with ±5 mV *ac* voltage amplitude after different stabilization period. Instrumental system consisted of Solartron 1287 potentiostat/galvanostat and Solartron FRA 1260 (Solartron Analytical, Farnborough, UK) controlled by ZPlot^®^ software ver. 3.5e (Scribner Associates, Southern Pines, NC, USA). ZView^®^ software ver. 3.5e (Scribner Associates, Southern Pines, NC, USA), based on complex non-linear least squares (CNLS) fit analysis [47], was utilized to model experimental data with χ^2^ values less than 5 × 10^−3^.

### 2.4. Computational Study

Density functional theory (DFT) quantum chemical calculations have been conducted using the Gaussian 09 program (revision D.01) [48]. The Truhlar’s M06 functional [49,50,51], the Pople’s 6-31+G(d,p) double-ξ basis set for H, C, O, N, P atoms, and the LANL2DZ basis set for the titanium (Ti) atoms [52] were utilized. The geometries were fully optimized using the 6-31+G(d,p) + LANL2DZ basis set. The vibrational frequency analysis at the same level of theory under the harmonic oscillator approximation to be true minima on the potential energy surface has been used to verify all calculated structures. To evaluate the water solvent effect, an implicit solvatation model based on density (SMD) has been employed [53]. The topological analysis of the charge density distibution was performed by the Bader’s quantum theory of atoms in molecules [54] by using AIMALL software (version 17.01.25) [55]. The (TiO_2_)_10_ nanocluster was used as a model for all possible molecular implant surface/alendronate interaction predictions [56,57]. Detail description of the modeling, as well as results (Tables S1–S4), are given in Supplementary Materials.

## 3. Results and Discussion

### 3.1. The Wetting Properties of Implant Samples

Changing of wetting properties due to the implant surface modification can confirm the successful coating formation. Besides, understanding the implant’s wetting properties is useful for predicting initial interactions between implant-bone and implant-surrounding medium that are crucial for the long-term stability of implant materials in the human body.

**Figure 2:**
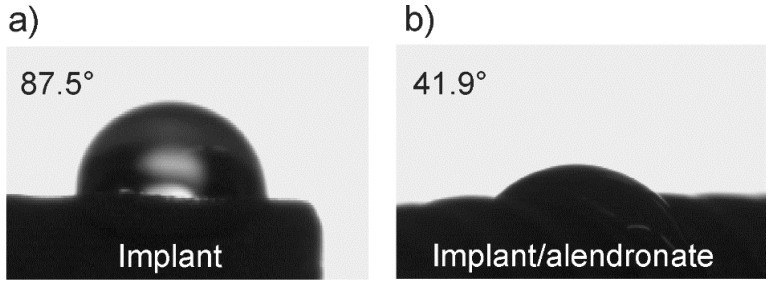
The water drops and contact angle values on (**a**) the as-received implant surface and (**b**) the alendronate-modified implant surface.

A water drop wetted unmodified and alendronate-modified implant surface completely different as can be seen from Figure 2. The contact angle value, *θ* measured on the unmodified implant surface (*θ =* 87.5 ± 2.28°) is twice higher than that measured on the alendronate-modified surface (*θ* = 41.9 ± 2.0°). Results for this commercially available titanium dental implant [58], as well as alendronate-modified titanium surfaces [30,32], are in agreement with literature data.

The obtained contact angle values point to important conclusions: (i) the alendronate coating was successfully prepared on the implant surface and (ii), due to the functionalization by alendronate molecules, nearly hydrophobic nature of the unmodified implant surface was changed to hydrophilic one, i.e., the modified implant surface reflects an enhanced wettability.

It is well-known that terminal functional groups at the outer interface (coating/water drop) determine surface polarity and wettability. The alendronate coating significantly changed the contact angle value of the implant, indicating a presence of a well-ordered coating in which hydrophilic functional groups, –NH_2_ and/or –PO_3_H, and/or –OH (originating from alendronate molecules, Figure 1b) affect wetting properties of the modified implant. A correlation with XPS and DFT results (see Section 3.2 and Section 3.3) will give a detailed insight into the mechanism of alendronate molecule bonding to the implant surface and contribute to the understanding of wetting properties.

It should be emphasized, for implant practical use, that, according to numerous in vivo and in vitro studies, a hydrophilic nature is one of desirable implant surface characteristics, which directly affects biological responses, such as protein adhesion, hard and soft tissue cell interactions, biofilm (bacterial) formation, as well as adhesion and differentiation of osteoblasts, and bone-building cells [31,35,59]. According to published data of in vitro studies, surfaces with water contact angle between 0° and 62° positively affect adhesion and differentiation of osteoblasts [35,60]. Therefore, hydrophilic coatings, such as alendronate coating, could directly influence on osteoblasts adhesion and consequently accelerate osseointegration process of dental implant. Future experiments that would determine a direct correlation between hydrophilicity with biological outcomes of alendronate-functionalized implants are challenging.

### 3.2. The Chemical Characterization of Implant Samples

**Figure 3:**
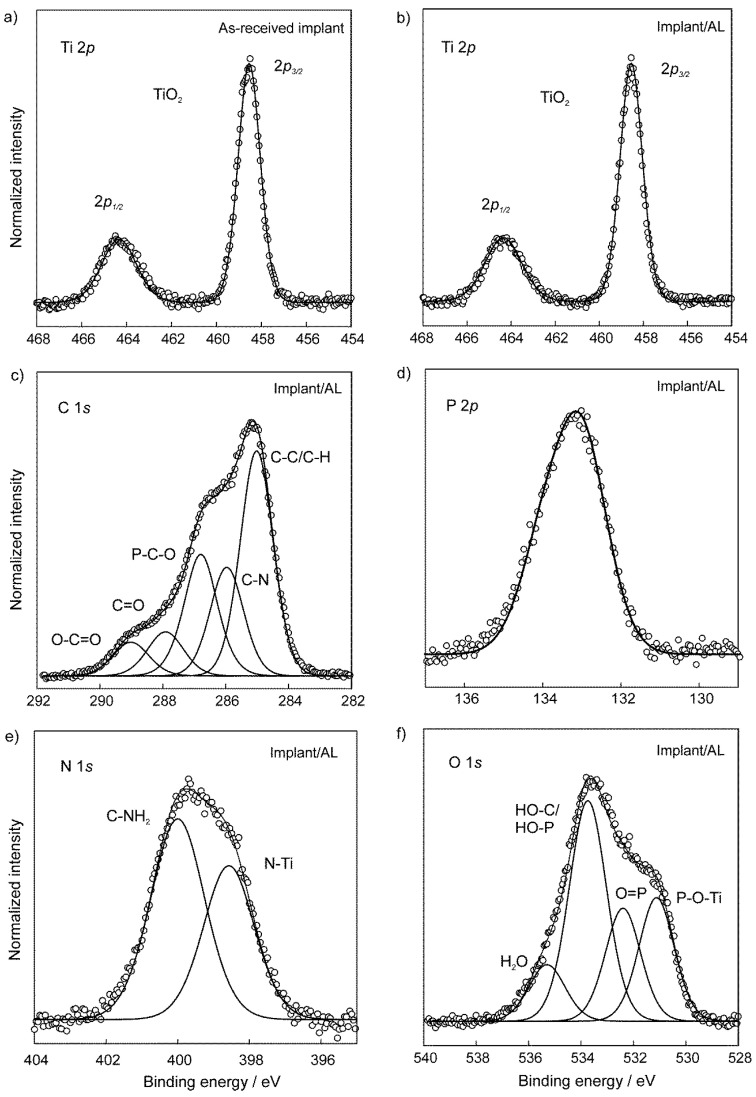
High-resolution XPS spectra (**a**) around Ti *2p* core-level of the as-received implant; (**b**) Ti 2*p;* (**c**) C 1*s*; (**d**) P 2*p*; (**e**) N 1*s*; (**f**) O 1*s* core-levels of the alendronate-modified implant surface (Implant/AL).

The information about chemical states and atomic bonding in the as-received implant and the implant modified with the alendronate coating was took out from chemical shifts in XPS measurements around core-levels of specific elements. The photoemission spectrum around Ti 2*p* core-level measured on the as-received implant (Figure 3a) shows a typical structure characteristic for TiO_2_ [61,62]. It consists of a spin-orbit doublet with the separation of 5.8 eV between the Ti 2*p*_3/2_ and Ti 2*p*_1/2_ peaks and the energy position of Ti 2*p*_3/2_ line at the BE of 458.5 eV. Therefore, the implant covered with the TiO_2_ layer represented a starting surface for functionalization with alendronate molecules.

After modification with the alendronate coating, the Ti 2*p* spectrum (Figure 3b) does not reveal any changes, confirming good chemical stability of the TiO_2_ layer. Detection of Ti signals from the underlying implant indicates that the alendronate coating is very thin and it is in agreement with DFT results (Figure S1, Supplementary Material). The XPS spectra around N 1*s*, C 1*s*, P 2*p*, and, O 1*s* core-levels, measured on the alendronate-modified implant demonstrate the successful bonding of alendronate molecules to the implant surface. First of all, the best fit of the photoemission around C 1*s* levels, shown in Figure 3c, requires five fitting components, related to the characteristic bonds in the alendronate molecule, namely the aliphatic C–C, C–N, and P–C–O bonds at BEs of 285.0 eV, 286.0 eV, and 286.8 eV, respectively [63,64]. Additional C=O and O–C=O bonds can be assigned to surface contaminations. On the other hand, a strong signal from phosphorus was detected in the XPS measurements, as shown in Figure 3d, for the P 2*p* emission. The spectrum was fitted with a spin-orbit doublet, where the P 2*p*_3/2_ peak is centered at the BE of 133.2 eV, while the separation of the P 2*p*_1/2_ component is shifted by only 0.9 eV. Results are consistent with already published data [33,35,63].

XPS data are useful for understanding the binding mechanism of the alendronate molecule onto the implant substrate. As can be seen from Figure 3e, the deconvolution of N 1*s* photoemission curve shows two distinct components at the BEs of 400.0 eV (with the relative atomic concentration fraction of 58%) and 398.5 eV (42%), respectively, which can be assigned to free C–NH_2_ bonds from the alendronate molecule and N atoms bonded to the implant (Ti–N bond), respectively [33]. On the other hand, the best fitting of the O 1*s* spectrum (Figure 3f) requires four components at BEs of 531.1 eV, 532.4 eV, 533.7 eV, and 535.3 eV. The peak at 531.1 eV (relative concentration fraction of 23%) is attributed to the formation of P–O–Ti bonds, and the peaks at 532.4 eV (21%) and 533.7 eV (45%) to the presence of P=O and H–O–C/H–O–P bonds, respectively [33,64,65,66]. In addition, a small contribution, observed at the BE of 535.3 eV, is most likely related to adsorbed water molecules [67].

The above photoemission results (Figure 3) confirm that the alendronate coating can be successfully obtained from aqueous solutions on dental implants by a simple method, such as self-assembly. Besides, the oxide, TiO_2_ layer is needed on the titanium-based implant for a successful chemical bonding of alendronate molecules through two possible bonding mechanisms, via the phosphonate (–PO_3_H) and amine (–NH_2_) functional group.

It is known that phosphonic acids, as well as bisphosphonates, can be bonded to the substrate in mono-/bi-/tridentate mode of bonding [31,35,43,68]. In the case of the sample studied in the present work, it is difficult to predict the phosphonate binding mode, since P–O and C–O binding energies are approximately the same (Figure 3f) [66], and sample contamination was also detected in C 1*s* and O 1*s* spectra (C=O, O–C=O in Figure 3c; H_2_O in Figure 3f). The correlation of XPS results with DFT findings (Section 3.3) will help to clarify possible binding modes of the phosphonate group of the alendronate molecule to the implant surface.

The presence of free –COH, –NH_2_, and –PO_3_H groups (Figure 3c,e,f) in the alendronate coating explains hydrophilicity of the alendronate-modified implant surface detected by contact angle measurements (Section 3.1). Namely, these free functional groups are positioned in the coating upper part (coating/water interface) and, therefore, can be sensed by the water drops.

### 3.3. The Mechanism of the Alendronate Coating’s Formation on the Implant Surface

The proposed mechanism of alendronate coating’s formation on the implant surface is based on findings of experimental and theoretical studies. The coating formation mechanism was investigated by theoretical simulations of implant surface/alendronate interactions employing quantum chemical calculations at the DFT. DFT results were correlated with experimental XPS findings. As confirmed by XPS (Section 3.2), the TiO_2_ layer is present on the implant surface; therefore, a suitable model, the (TiO_2_)_10_–alendronate, was chosen for theoretical hypothetical simulations of all possible molecular implant surface/alendronate interactions. For computational efficiency, the small (TiO_2_)_10_ nanocluster served for cluster modeling, used by Qu and Kroes [57].

The correlation of the result of calculated Gibbs free energies and energies for all (TiO_2_)_10_–alendronate binding interactions (as theoretical simulations of implant surface/alendronate interactions) with results of surface-analytical methods (above discussed XPS and contact angle results) leads to the conclusions regarding probable molecular implant surface/alendronate interactions. Calculated Gibbs free energies of (TiO_2_)_10_–alendronate interactions revealed the spontaneous formation of the alendronate coating (ΔG*_INT_ < 0), indicating two possible ways of alendronate molecules bonding to the implant surface, via amine (–NH_2_) and/or phosphonate (–PO_3_H) group. All possible binding sites on (TiO_2_)_10_ clusters were calculated by DFT and are shown in Table S2.

**Figure 4:**
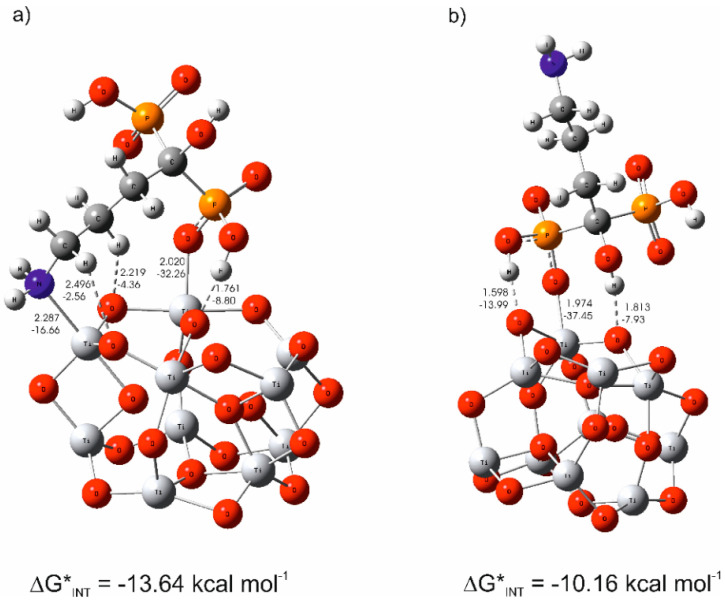
(**a**) The most stable structure and (**b**) less stable structure of the (TiO_2_)_10_–alendronate, predicted by density functional theory (DFT), with calculated bond distances in Å and bond energies in kcal mol^−1^.

The DFT calculations showed that the alendronate simultaneously participates in interactions through both phosphonate (Ti–O) and amine (Ti–N) groups in the most stable structure of (TiO_2_)_10_–alendronate (Figure 4a). As a consequence of (TiO_2_)_10_–alendronate interactions, a free electron pair on the nitrogen atom from an amine group was involved in the formation of a new coordinate bond with titanium (Ti–N; *d*_Ti–N_ = 2.287 Å, *E*_Ti–N_ = −16.66 kcal mol^−1^) being accompanied by two weaker C–H∙∙∙O hydrogen bonds (*E*_O∙∙∙H_ ranges from −2.56 to −4.36 kcal mol^−1^, *d*_O–H_ ranges from 2.219 to 2.496 Å; see Figure 4a). The critical point of the Ti–N bond was characterized by ∇^2^*ρ(rc)* > 0 and *H(rc)* < 0; therefore, the Ti–N bond is attributed to an intermediate type of interaction. However, additional stabilization of the most stable (TiO_2_)_10_–alendronate structure was accomplished simultaneously through the phosphonate group of the alendronate. Herein, a free electron pair on the oxygen atom from the phosphonate group of alendronate was involved in strong bonding with titanium (*d*_Ti–O_ = 2.020 Å, *E*_Ti–O_ = −32.24 kcal mol^−1^), being accompanied by O–H∙∙∙O hydrogen bond between the hydrogen atom from the hydroxyl group of same phosphonate branch and the oxygen atom in (TiO_2_)_10_ cluster (*d*_O∙∙∙H_ = 1.761 Å, *E*_O∙∙∙H_ = −8.80 kcal mol^−1^ see Figure 4a). The Ti–O bond is characterized as an ionic type of interaction according to ∇^2^*ρ(rc)* > 0 and *H(rc)* > 0. When two strong (Ti–N and Ti–O) bonds, one stronger hydrogen O–H∙∙∙O and two weaker C–H∙∙∙O hydrogen, were formed, the free energy of (TiO_2_)_10_–alendronate interactions was released (ΔG*_INT_ = −13.64 kcal mol^−1^). Due to the high affinity of alendronate towards (TiO_2_)_10_ surface based on its simultaneous participation in bond formation via both branches, phosphonate and amine, a high coverage level is achieved, and high free energy is released.

Besides the most stable (TiO_2_)_10_–alendronate structure (Figure 4a), the theoretical study was extended to another stable (TiO_2_)_10_–alendronate structure (Figure 4b), energetically closely competitive exhibiting ΔG*_INT_ = −10.16 kcal mol^−1^, in detail, being for 3.48 kcal mol^−1^ less stable than the most stable structure. The observed DFT results clearly indicate a different motif and possible ways of alendronate molecules bonding to the implant surface compared to the most stable structure. Herein, in a different manner, the binding was accomplished solely through the phosphonate group, which involves coordinate Ti–O bonding (*d*_Ti–O_ = 1.974 Å, *E*_Ti–O_ = 37.45 kcal mol^−1^ and two hydrogen bonds (O–H∙∙∙O). Since ∇^2^*ρ(rc)* > 0 and *H(rc)* > 0, the Ti-O bond represents an ionic type of interaction. As shown in Figure 4b, one O–H∙∙∙O hydrogen bond was accomplished between the hydrogen atom from the hydroxyl group of same phosphonate group and the oxygen atom in the (TiO_2_)_10_ cluster (*d*_O∙∙∙H_ = 1.598 Å, *E*_O∙∙∙H_ = −13.99 kcal mol^−1^), another between the hydrogen atom from the hydroxyl group of the C–O–H branch and the oxygen atom in the (TiO_2_)_10_ cluster (*d*_O∙∙∙H_ = 1.813 Å, *E*_O∙∙∙H_ = −7.93 kcal mol^−1^; see Figure 4b). It is important to emphasize the presence of a free NH_2_ group in the alendronate molecule, oriented in the upper part of the (TiO_2_)_10_–alendronate structure, as can be seen in the motif in Figure 4b. When one strong Ti–O and two hydrogen (O–H∙∙∙O) bonds were formed, the free energy of (TiO_2_)_10_–alendronate interactions was released (ΔG*_INT_ = −10.16 kcal mol^−1^).

Most likely, both of the above-discussed thermodynamically most stable (TiO_2_)_10_–alendronate structures would become energetically competitive, providing pronounced dynamics of self-assembling coating process. Spontaneous formation of both above-mentioned most stable (TiO_2_)_10_–alendronate structures plays an important role in fully clarifying the coating’s formation mechanism. It was established that the binding is more exergonic in case of direct interaction of alendronate with implant surface via two strong bonds, Ti–N and Ti–O, through simultaneous participation common to both phosphonate and amine branches (Figure 4a), in comparison to the binding accomplished solely through the phosphonate group via the Ti–O bond (Figure 4b). Considering the DFT results, one might conclude that the alendronate coating’s formation process occurs mainly through the most stable (TiO_2_)_10_–alendronate structure (Figure 4a), where alendronate simultaneously participates via both bonding groups, phosphonate (Ti–O) and amine (Ti–N), rather than solely through phosphonate group (Figure 4b).

However, it was not possible to clearly establish the mechanism of the alendronate coating’s formation on the implant surface solely from the DFT results. A reliable experimental confirmation was needed. Therefore, DFT results were correlated with experimental results of wetting properties and XPS measurements, and these findings enabled to suggest general conclusions regarding the alendronate coating formation mechanism on the oxide-covered (TiO_2_) implant surface.

Wetting properties changed upon the alendronate functionalization from closely hydrophobic (as-received implant, *θ* = 87.5 ± 2.2°) to hydrophilic (alendronate-modified surface, *θ* = 41.9 ± 2.0°). The presence of the TiO_2_ layer on the as-received implant, confirmed by XPS, reflects a hydrophobic character of the investigated surface. On the other hand, a hydrophilic nature of the alendronate-modified surface can be explained by DFT and XPS findings. Namely, two above-discussed thermodynamically stable (TiO_2_)_10_–alendronate structures (Figure 4a,b) are, according to DFT calculations, energetically competitive and most probably both occur simultaneously during the formation process. This conclusion, verified by the XPS (Section 3.2), which detected Ti–N and Ti–O linkages but also free –NH_2_ and –COH groups, is in agreement with both investigated (TiO_2_)_10_–alendronate structures (Figure 4a,b). In the case of alendronate bonding via the phosphonate group, DFT results indicate most probably a bidentate-bonding mode (1 coordinate bond + 1 hydrogen bond). Free hydroxyl and amine groups, as well as the second, unbounded –PO_3_H group of the alendronate molecule, are oriented in the coating’s upper part, and they are responsible for the hydrophilic character observed by contact angle measurements.

To sum up, results of all techniques used in the present study confirm formation of the alendronate coating on the oxide-covered implant surface via both phosphonate (–PO_3_H) and amine (–NH_2_) functional groups. It is important to emphasize that the resulting hydrophilic character of the dental implant surface is essential from the osseointegration point of view, and it could be obtained by self-assembly of bioactive molecules, like alendronate.

### 3.4. The Electrochemical Chracterization of Implant Samples

**Figure 5:**
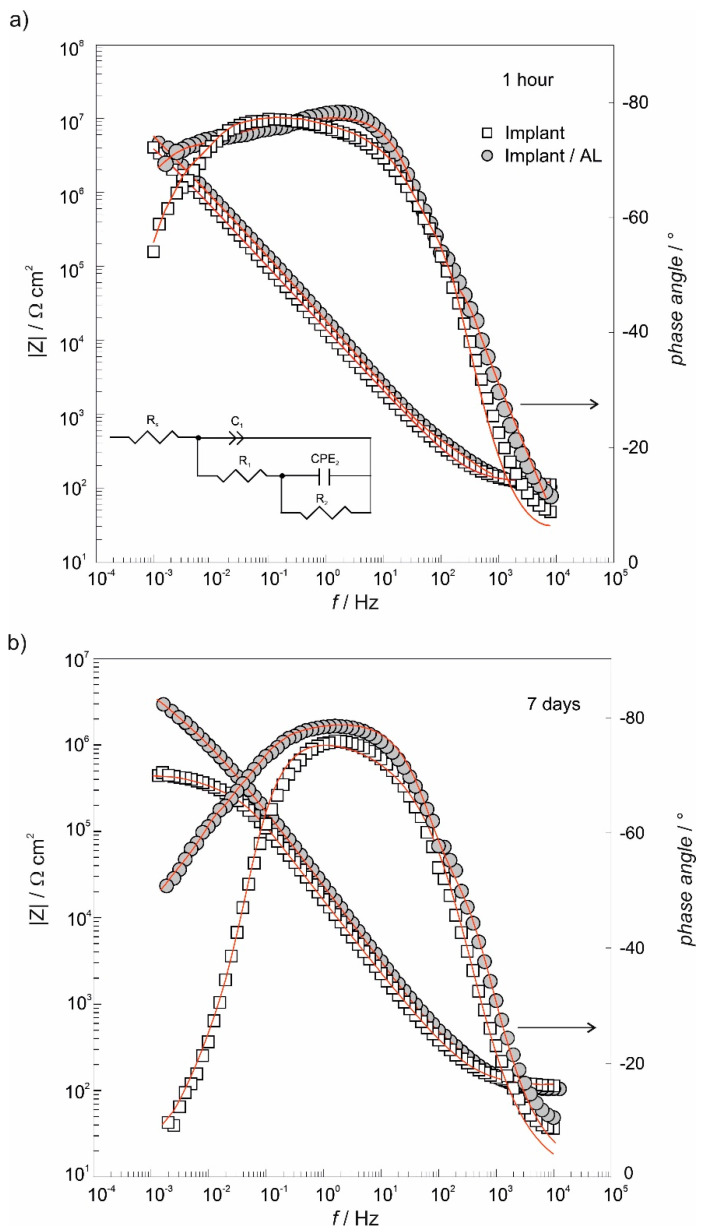
Electrochemical impedance spectroscopy (EIS) responses of as-received implant (Implant) and alendronate-coated implant (Implant/AL) samples recorded after (**a**) 1 h- and (**b**) 7 days-stabilization period at the open circuit potential in the artificial saliva, pH = 6.8. Symbols: the experimental data; solid lines: the modeled data.

The electrochemical stability of the alendronate-modified implant after 1 h- and 7 days-exposure to an artificial saliva solution was investigated in vitro by an electrochemical impedance spectroscopy technique. Results, in the form of Bode plots, are displayed in Figure 5. In the case of the as-received implant after a 1-h immersion period, the dependence phase angle against log *f* in the middle frequency range is quite wide (Figure 5a) due to the TiO_2_ presence on the implant surface as was confirmed by XPS (Figure 3a). At the same time, the dependence log |*Z*| against log *f* achieves a high value close to 10^7^ Ω cm^2^ and points to a protective/barrier role of the TiO_2_ layer in the corrosion protection of the as-received implant in an aggressive saliva solution.

The formation of the alendronate coating on the implant/TiO_2_ interface influenced the structure of the electrified implant/TiO_2_/electrolyte interface (Figure 5a). This is especially visible in the dependence phase angle against log *f* that is wider in the middle-frequency range compared to the corresponding response of the as-received implant. The structural-sensitive phase angle reflected coating’s microstructural transformations (implant/TiO_2_/alendronate) after modification of the implant/TiO_2_ surface.

Although the TiO_2_ layer offered a good anti-corrosion protection to the implant (10^7^ Ω cm^2^; Figure 5a), |*Z*| values at low frequencies, obtained after 7 days-immersion period, decreased more than one order of magnitude compared to the response recorded after a 1-h immersion period, indicating the deterioration of anti-corrosion protection. On the other hand, from Figure 5b, a positive impact of the alendronate coating on implant protective properties is clearly visible. The coating presence on the implant surface extended anti-corrosion protection up to 7 days; the phase angle versus log *f* dependence is wider and |*Z*| values are higher in comparison to |*Z*| values of the as-received implant (Figure 5b). This behavior is very important for the successful long-life applications of dental implants in aggressive environment, like the human body.

The quantification of the anti-corrosion protection was performed by modeling of EIS data (Figure 5) using the electrical equivalent circuit (EEC) presented as *R*_s_(*C*_1_(*R*_1_(*CPE*_2_*R*_2_))); see the inset in Figure 5a. In the case of the as-received implant/artificial saliva interface, the chosen model represents the oxide film of the bi-layered structure formed on the titanium [69,70]. *R*_s_ is the electrolyte resistance and (*R*_1_*C*_1_) time constant, in the high/middle-frequency region, is associated with the outer part of the oxide layer with *R*_1_ as the resistance and *C*_1_ representing the capacitance of the oxide outer part. The second time constant, (*R*_2_*CPE*_2_), in the low-frequency region, is correlated to the inner part of the oxide layer with *CPE*_2_ as the capacitance and *R*_2_ as the resistance of the inner part of the oxide.

In the case of the alendronate-modified implant, the high/middle-frequency time constant (*R*_1_*C*_1_) represents surface coating’s (alendronate over TiO_2_ layer) resistance and capacitance, respectively, while the low-frequency time constant (*R*_2_*CPE*_2_) is connected with resistance and capacitance of coating’s structural defects [71]. Calculated values can be found in Table 2.

Microscopic inhomogeneities of investigated interfaces [72,73] were the reason for using the constant phase element (CPE) instead of a capacitor. The impedance of the constant phase element is defined as *Z_CPE_* = [*Q*(j*ω*)n]^−1^, where ω is the angular frequency, and *Q* is the frequency-independent constant. If the *CPE* exponent n has a value, n = 1, the *CPE* can be replaced by a pure capacitor, *C*. [72]. The Brug’s Equation (1) [73] was used to calculate capacitance values, *C*, which are also presented in Table 2.

*C* = *Q*^1/n^ [*R*_s_^−1^ + *R*^−1^]^(n−1)/n^ (1)

As can be seen from resistance values in Table 2, the oxide inner part (implant/oxide interface) is responsible for good barrier properties (*R*_2_ is high compared to *R*_1_) of the as-received implant during 1 h-immersion in the artificial saliva (Figure 5a). Due to the imperfect structure of the outer oxide part by longer immersion in the saliva up to 7 days, water/ions attack deeper in the oxide structure was enabled and, consequently, resistance values decreased. The polarization resistance, *R*_p_ [74], which is the sum of both *R*_1_ and *R*_2_ values, defines overall corrosion protection of the TiO_2_ layer provided to the underlying titanium.

As mentioned before, the coating (alendronate + TiO_2_) presence improves anti-corrosion protection of the investigated implant, confirmed also by results in Table 2. Alendronate molecules obviously filled in oxide surface imperfections, and overall corrosion resistivity was increased. Since the time constant in the low-frequency range (*R*_2_*CPE*_2_), representing microstructural defects, appeared during the formation process, their influence was, according to high resistance *R*_2_ value, almost negligible after a short immersion period of the modified implant in the saliva solution. However, the effect of microstructural defects on coating’s overall resistivity is clearly visible in the decreased *R*_2_ value after a prolonged immersion period of 7 days. The explanation of such behavior lies in surface hydrophilicity of the alendronate coating determined by contact angle measurements (Section 3.1). Terminal groups at the outer alendronate coating interface (coating/artificial saliva), free –NH_2_, –COH, and –PO_3_H groups, determined by DFT and XPS (Figure 3 and Figure 4), affected wettability of the modified surface and were responsible for hydrophilic character. Hydrophilic surfaces interact easily with water molecules and other ions, especially Cl^−^ ions in artificial saliva solution through dipole-dipole and ion-dipole interactions [44]. Structural rearrangement occurs, and the alendronate coating structure transforms into structures that favor ions/water penetration into the coating. The result of this rearrangement is an increase of defect density and size, while water/ions become trapped into the coating’s structure, as reflected in a decreased coating’s resistivity.

Although the as-received titanium implant shows good anti-corrosion properties, functionalization by alendronate molecules provides an additional barrier during its exposure to the artificial saliva solution. The alendronate coating is stable on the implant surface during the 7 days-immersion period due to the coordinate Ti–O and Ti–N bonding (XPS and DFT results) being additionally stabilized by the formation of the hydrogen bonds (DFT results). Beside good anti-corrosion protection, alendronate molecules, due to their known positive influence on the bone system, make the implant surface potentially more bioactive for a faster osseointegration process. The assumption is that the alendronate coating will attract bone-building cells, osteoblasts to the implant surface, and thus accelerates the osseointegration process that will be tested in the continuation of investigations.

## 4. Conclusions

Correlation of results of all techniques used in this study, both theoretical and experimental, provides strong indication of the preparation of stable and chemically bonded alendronate coating on the titanium dental implant by a simple immersion procedure.

Chemical characterization of the coating was obtained from XPS measurements. The coated implant surface is covered with an inner TiO_2_ layer and an outer alendronate layer. Alendronate molecules are bonded to the TiO_2_-covered implant surface via amine (–NH_2_) and phosphonate (–PO_3_H) functional group. Simultaneously, a significant part of (–NH_2_), as well as (–COH), groups of alendronate molecules remains free and determines surface properties of the modified implant.

Free –COH, –NH_2_, as well as unbounded –PO_3_H, groups of the alendronate molecule are presented at the alendronate coating/water drop interface and responsible for a hydrophilic character of the modified implant surface (*θ =* 41.9 ± 2.0°).

XPS and wettability results correlate well with the results of DFT calculations. According to the DFT findings, formation of the alendronate coating occurs most probably through two energetically competitive structures, one in which the alendronate molecule is bound to the implant surface via amine (–NH_2_) and phosphonate (–PO_3_H) groups (ΔG*_INT_ = −13.64 kcal mol^−1^) and the other in which the alendronate molecule is bound solely via phosphonate (–PO_3_H) group (ΔG*_INT_ = −10.16 kcal mol^−1^).

Both structures include the additional formation of hydrogen bonds, and this kind of bonding provides very good coating stability during 7-days exposure of the modified implant to the artificial saliva solution (overall resistivity, *R* ≈ 5.9 MΩ cm^2^).

From a practical point of view, a low-cost and simple procedure can be used for producing a stable, corrosion-resistant, and, at the same time, potentially bioactive coating, which could prolong a life cycle of titanium dental implants in the body.

Due to well-known bioactivity of the alendronate as a drug for bone diseases, there is a need for evaluation of its bioactivity as a surface coating. Therefore, a part of future work will be focused on in vitro investigations of osteoconductivity and bioactivity of the alendronate-modified dental implants.

## Figures and Tables

**Table 1:** Chemical composition (wt %) of titanium c.p. grade 2 [41].

Element	N	C	O	Fe	H	Ti	Other
wt %	0.03	0.10	0.25	0.30	0.0155	Balance	0.4

**Table 2:** Impedance parameters calculated from EIS data (Figure 5) for the as-received implant (Implant) and the alendronate-modified implant (Implant/AL).

	*R*_s_/ Ω cm^2^	*C*_1_/ µF cm^−2^	*R*_1_/ Ω cm^2^	*Q*_2_·10^6^/ Ω^−1^ cm^−2^ s^n1^	n_2_	*C*_2_/ µF cm^−2^	*R*_2_/ MΩ cm^2^
*Exposure time of 1 h*							
Implant	111	3.02	760	5.16	0.850	1.38	9.90
Implant/AL	109	2.00	307	9.21	0.820	2.03	39.0
*Exposure time of 7 days*							
Implant	123	2.71	307	9.21	0.810	1.88	0.44
Implant/AL	109	1.98	302	7.23	0.795	1.15	5.88

## Supplementary Materials

The following are available online at https://www.mdpi.com/1996-1944/13/14/3220/s1, Figure S1: The thickness of the most stable (TiO_2_)_10_–alendronate(1) and (TiO_2_)_10_–alendronate(2) species, Table S1: Formation of the most stable (TiO_2_)_10_−alendronate(1) and (TiO_2_)_10_−alendronate(2) species^(a)^. Standard state (1M) free energies of interaction Δ_r_*G**_INT_ computed by using the SMD solvation model at the M06/6-311++G(2df,2pd) + LANL2DZ// M06/6-31+G(d,p) + LANL2DZ level of theory, Table S2: Bond lengths (*d*), energies I and QTAIM properties of the selected bonds in the most stable (TiO_2_)_10_–alendronate(1) and (TiO_2_)_10_–alendronate(2), Table S3: Total electronic energy, *E*^Tot^_soln_, obtained at the SMD/M06/6-311++G(2df,2pd)+ LANL2DZ//SMD/M06/6-31+G(d,p) + LANL2DZ level of theory, thermal correction to the Gibbs free energy, ΔG*_VRT,soln_, obtained at the SMD/M06/6-31+G(d,p) + LANL2DZ level of theory, and total free energy, G*_X_, (G*_X_ = *E*_Totsoln_ + ΔG*_VRT,soln_) in water media of the investigated species (all energies in hartree), Table S4: Cartesian coordinates of the calculated syste–s-alendronate on the surface of (TiO_2_)_10_ cluster in water media.
