# Supplementary material for: A New Insight into Coating’s Formation Mechanism Between TiO2 and Alendronate on Titanium Dental Implant
Source: Materials (Basel). 2020 Jul 20;13(14):3220. doi: 10.3390/ma13143220 (PMC7411690; doi:10.3390/ma13143220)
Supplement: Supplementary file 1 [file materials-13-03220-s001.pdf]

materials

Supplementary Materials

Computational modeling

All calculations were performed by means of quantum chemical calculations at the density functional theory (DFT) level using the Gaussian 09 program (revision D.01) [1]. The $(TiO_2)_n$ clusters were employed, proposed by Allard et al. [2] and Qu and Kroes [3] with n = 10.

The M06 functional designed by the Truhlar's group, which provides very accurate thermodynamic parameters, being particularly successful in nonbonding interactions treatment, has been selected [4–6]. The 6-31+G(d,p) + LANL2DZ mixed basis set has been utilized. The Pople's 6-31+G(d,p) double-ξ basis set was chosen for O, H, C, N, P atoms and the LANL2DZ basis (LANL2 pseudopotential for inner electrons and its associated double-ξ basis set (DZ)) was used for the transition-metal (Ti) atoms [7]. This gave rise to the M06/6-31+G(d,p) + LANL2DZ model utilized for geometry optimization which has been frequently used for studies of transition-metal containing systems. The geometric structures of the molecules were optimized by minimizing energies with respect to all geometrical parameters without imposing any molecular symmetry constraints and using a tight convergence condition. The Berny algorithm using redundant internal coordinates was employed. Frequency calculations were made under the harmonic approximation on all the optimized structures at the same level of theory with no scaling in order to confirm that the structures correspond to the true minima, meaning that no imaginary frequencies were present, as well as to extract thermal Gibbs free energy corrections. The final single point energies were obtained using a highly flexible 6-311++G(2df,2pd) basis set for the O, H, C, N, P atoms, while the same LANL2DZ ECP type basis set for titanium atoms was employed.

The self- consistent field (SCF) calculations were conducted under a tight condition imposing the threshold value of 10^{-8} hartree to total energy difference during the iteration process. The integration grid was set to FineGrid having 75 radial shells and 302 angular points per shell. The 2-electron integral accuracy was set to 10^{-13}. The FoFCou algorithm with NoSymm option was utilized. All the geometry optimizations, frequency calculations and single point energy evaluations were performed by taking solvent effects into account. To evaluate the bulk solvent effects (water, ε = 78.3553 at 25°), the implicit SMD polarizable continuum solvation model [8] has been employed. It represents a very practical approach to simulate the solvation environment and determine the effect of the medium on the structure and stability of solutes in a solution. The conformational space was manually sampled for the $(TiO_2)_{10}$—alendronate taking into account various donor and acceptor sites of the $(TiO_2)_{10}$ cluster and alendronate molecule. Two thermodynamically most stable $(TiO_2)_{10}$—alendronate structures are reported here. The starting structure of the alendronate was taken from the literature [9] and modified subsequently to a dianionic form.

The interaction Gibbs free energies, ΔG^{*}_{INT} were computed as the difference between the total free energy (G^{*}_{AB}) of the $(TiO_2)_{10}$—alendronate structure and the sum of the total free energies (G^{*}_{A} + G^{*}_{B}) of the associating units A and B ($(TiO_2)_{10}$ cluster and alendronate molecule) using the supramolecular approach (Equation (1)):

$$\Delta G^{*}_{INT,AB} = G^{*}_{AB} - G^{*}_{A} - G^{*}_{B} \quad (1)$$

The species total free energy in the liquid was calculated using the expression (Equation (2)):

$$G^{*}_{X} = E^{Tot}_{soln} + \Delta G^{*}_{VRT,soln} \quad (2)$$

where E^{Tot}_{soln} corresponds to the basic energy of a density functional theory calculation using the SMD model, while $\Delta G^{*}_{VRT,soln}$ encompasses vibrational, rotational and translational contribution to the solution free energy, being computed by applying the ideal gas partition functions to the frequencies calculated in the dielectric medium and the 1M standard state. A more negative value of the binding energy implied the more stable formed species. No BSSE correction of binding energies has been applied.

The topological analysis of the charge density distribution using the Bader's quantum theory of atoms in molecules (QTAIM) [10] was performed by employing AIMALL software package [11] using the SMD/M06/6-31+G(d,p) + LANL2DZ wave function obtained from optimization. Within the QTAIM analysis the electron density was analysed for two major characteristics: (a) the existence of critical points (CPs), where electron density exhibits maximum, minimum, or a saddle point in space, and (b) for the bond paths [12], the maximum electron density line connecting two interacting atoms in the energetic minimum structure (the two atoms are bonded). The point of the electron density minimum value along that line called the bond critical point (BCP) and values of topological parameters, like electron density $\rho(r_c)$, Laplacian $\nabla^2\rho(r_c)$, electronic kinetic energy $G(r_c)$, electronic potential energy density $V(r_c)$, total energy density $H(r_c)$ at that point explain interatomic interaction features. $\nabla^2\rho(r_c) < 0$ indicates locally concentrated charge density, while locally depleted charge density is indicated by $\nabla^2\rho(r_c) > 0$. The chemical bond nature can be described qualitatively concerning signs and values of the electron density Laplacian $\nabla^2\rho(r_c)$ and the electron energy density $H(r_c)$ at the bond critical point according to following criteria.

The interactions characterized by $\nabla^2\rho(r_c) < 0$ and $H(r_c) < 0$ (shared interaction) are characteristic for weakly polar and nonpolar covalent bonds. On the other hand, $\nabla^2\rho(r_c) > 0$ and $H(r_c) > 0$ (closed shell interactions) point to ionic bonds, weak hydrogen bonds, and van der Waals interactions. The intermediate interactions which includestrong hydrogen bonds and most of the coordinate bonds are characterized by $\nabla^2\rho(r_c) > 0$ and $H(r_c) < 0$ [13,14]. A very high negative value of the $\nabla^2\rho(r_c)$ is an indication of a strong covalent bond, while a high positive value corresponds to a strong noncovalent bond. The energies of the coordinate bonds (Ti—O, Ti—N) and of other intermolecular hydrogen bonds have been calculated by the Espinosa's Equation (3) [15]:

$$E = 0.5\ V(r_c) \quad (3)$$

where E is the bond energy (a.u.), and $V(r_c)$ is potential energy density (a.u.) at the corresponding critical point. The Espinosa's relationship is widely used for the energy estimation of different types of hydrogen [16,17], van der Waals [18], coordinate [19,20], and homopolar bonds [21].

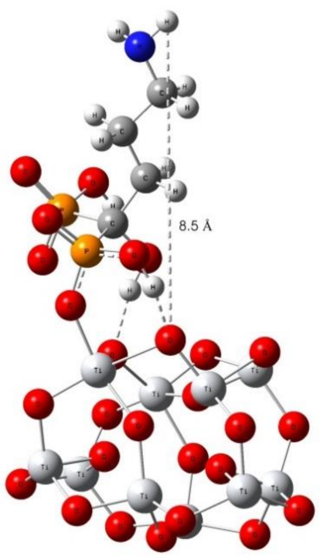	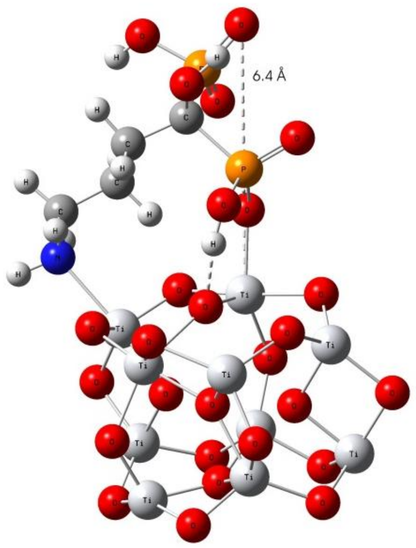
$(TiO_2)_{10}$−alendronic-acid-anion (**1**)	$(TiO_2)_{10}$−alendronic-acid-anion (**2**)

Figure S1. The thickness of the most stable $(TiO_2)_{10}$−alendronate (**1**) and $(TiO_2)_{10}$−alendronate (**2**) species.

Table S1. Formation of the most stable $(TiO_2)_{10}$–alendronate (**1**) and $(TiO_2)_{10}$–alendronate (**2**) species [a]. Standard state (1M) free energies of interaction $\Delta_r G^*_{INT}$ computed by using the SMD solvation model at the M06/6-311++G(2df,2pd) + LANL2DZ// M06/6-31+G(d,p) + LANL2DZ level of theory.

**Species**	**$\Delta_r G^*_{INT}$/ kcal mol^{-1}**
$(TiO_2)_{10}$–alendronic-acid-anion (**1**)	−13.64
$(TiO_2)_{10}$–alendronic-acid-anion (**2**)	−10.16

[a] According to the reaction: $(TiO_2)_{10}$ + Alendronate→$(TiO_2)_{10}$–alendronate.

Table S2. Bond lengths (d), energies (*E*) and QTAIM properties of the selected bonds in the most stable $(TiO_2)_{10}$–alendronate (**1**) and $(TiO_2)_{10}$–alendronate (**2**).

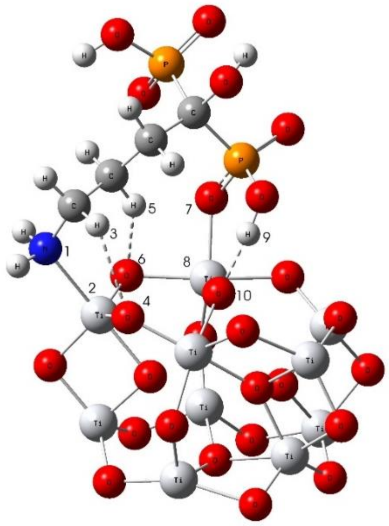

$(TiO_2)_{10}$–alendronate (**1**)

**Bond**	**d/Å**	**$\rho(r_c)/e{\times}a_0^{-3}$**	**$\nabla^2\rho(r_c)/e{\times}a_0^{-5}$**	**$V(r_c)$/au**	**$G(r_c)$/au**	**$H(r_c)$/au [a]**	**E/kcal mol^{-1} [b]**
N(1)—Ti(2)	2.287	5.395×10^{-2}	0.1657	−0.0531	0.0473	−0.0058	−16.66
H(3)—O(4)	2.496	1.141×10^{-2}	0.0406	−0.0082	0.0092	0.0010	−2.56
H(5)—O(6)	2.219	1.819×10^{-2}	0.0548	−0.0139	0.0138	−0.0001	−4.36
O(7)-Ti(8)	2.020	7.899×10^{-2}	0.4152	−0.1028	0.1033	0.0005	−32.26
H(9)-O(10)	1.761	3.710×10^{-2}	0.1150	−0.0281	0.0284	0.0003	−8.80

[a] $H(r_c) = V(r_c) + G(r_c)$, [b] $E = 0.5 \times V(r_c)$.

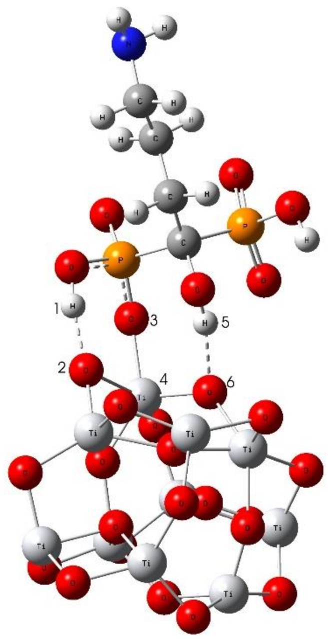

$(TiO_2)_{10}$–alendronate (**2**)

Bond	d/Å	$\rho(r_c)/e \times a_0^{-3}$	$\nabla^2\rho(r_c)/e \times a_0^{-5}$	$V(r_c)$/au	$G(r_c)$/au	$H(r_c)$/au [a]	E/kcal mol^{-1} [b]
H(1)—O(2)	1.598	5.315×10^{-2}	0.1690	−0.0446	0.0434	−0.0012	−13.99
O(3)—Ti(4)	1.974	8.723×10^{-2}	0.4894	−0.1194	0.1209	0.0015	−37.45
H(5)-O(6)	1.813	3.316×10^{-2}	0.1022	−0.0253	0.0254	0.0002	−7.93

[a] $H(r_c) = V(r_c) + G(r_c)$, [b] $E = 0.5 \times V(r_c)$.

Table S3. Total electronic energy, E^{Tot}_{soln}, obtained at the SMD/M06/6-311++G(2df,2pd) + LANL2DZ//SMD/M06/6-31+G(d,p) + LANL2DZ level of theory, thermal correction to the Gibbs free energy, $\Delta G^{*}_{VRT,soln}$, obtained at the SMD/M06/6-31+G(d,p) + LANL2DZ level of theory, and total free energy, G^{*}_{X}, ($G^{*}_{X} = E^{Tot}_{soln} + \Delta G^{*}_{VRT,soln}$) in water media of the investigated species (all energies in hartree).

Species	E^{Tot}_{soln}	$\Delta G^{*}_{VRT,soln}$	G^{*}_{X}
$(TiO_2)_{10}$	−2087.42891	0.01633	−2087.41258
Alendronate	−1423.53070	0.13691	−1423.39379
$(TiO_2)_{10}$–alendronate (**1**)	−3511.01315	0.18505	−3510.82810
$(TiO_2)_{10}$–alendronate (**2**)	−3511.00512	0.18257	−3510.82255

Table S4. Cartesian coordinates of the calculated systems-alendronate on the surface of $(TiO_2)_{10}$ cluster in water media.

$(TiO_2)_{10}$

**O**	0.039305	−0.015425	−0.096029
**Ti**	−0.142229	−0.016449	1.909261
**O**	1.643979	−0.081708	2.225577
**Ti**	2.588875	−1.632763	2.701598
**O**	1.385534	−2.340195	0.813656
**Ti**	0.500223	−1.634302	−0.700172
**O**	−0.835925	−2.909901	−0.425808
**Ti**	−0.717366	−3.425701	1.376934
**O**	−0.038901	−3.603219	3.256275
**Ti**	1.187160	−4.492811	4.427591
**O**	2.039784	−3.483926	5.822923
**Ti**	2.122282	−1.690467	5.604155
**O**	0.976851	−1.661306	4.177928
**Ti**	−0.446984	−0.773519	4.936094
**O**	−0.647342	0.552109	3.713348
**Ti**	−1.574243	−3.883261	4.129682
**O**	−2.165108	−4.078557	2.176736
**O**	0.736288	−0.670242	6.371498
**O**	−1.639573	−2.241342	4.926268
**Ti**	2.181085	−5.041926	1.774874
**O**	3.218363	−4.444322	0.285745
**Ti**	3.032921	−2.661180	−0.012552
**O**	2.183516	−1.956117	−1.502171
**O**	0.557956	−4.947496	1.108874
**O**	−0.982160	−1.540559	1.964775
**O**	−0.519545	−4.933019	5.194947
**O**	1.753850	−5.925654	3.534382
**O**	2.454572	−3.626436	2.916724
**O**	3.851642	−1.709565	1.363512
**O**	3.445548	−1.293750	4.274371

Alendronate

**C**	−0.003769	−0.046902	−0.010445
**P**	−0.838990	1.544174	−0.512918
**O**	−2.127865	1.650306	0.275468
**O**	0.083741	−0.033716	1.423615
**C**	1.443797	−0.160454	−0.489105
**C**	1.707612	−0.239803	−1.985748
**C**	3.177793	−0.488024	−2.259126
**N**	3.449527	−0.506996	−3.697171
**P**	−1.069513	−1.509859	−0.458973
**O**	−1.504968	−1.443287	−1.905748
**O**	−2.106581	−1.640104	0.638488
**O**	−0.065610	−2.792071	−0.243663
**O**	0.168322	2.673848	0.134606
**O**	−0.869104	1.726785	−2.015911
**H**	1.865262	−1.053068	−0.002001
**H**	1.993433	0.697570	−0.071558
**H**	1.115682	−1.048470	−2.444727
**H**	1.400485	0.690234	−2.481692
**H**	3.771988	0.317269	−1.806079
**H**	3.482559	−1.420841	−1.753139
**H**	−0.802369	−0.230872	1.771015
**H**	2.942275	−1.286431	−4.113043
**H**	4.435472	−0.705592	−3.848846
**H**	0.899671	2.884322	−0.465506
**H**	0.497597	−2.953087	−1.016039

$(TiO_2)_{10}$—alendronate (1)

**C**	−0.160091	−6.233433	1.165208
**O**	−0.873480	−5.973268	−0.049338
**C**	0.904564	−7.270466	0.802793
**C**	1.798312	−7.729077	1.941990
**C**	2.728128	−8.854334	1.536866
**N**	3.345879	−9.466360	2.736754
**Ti**	1.871866	−10.498133	4.149168
**O**	1.190332	−9.000501	4.787222
**Ti**	−0.849380	−8.981525	4.761221
**O**	−1.037250	−10.080029	3.450357
**Ti**	−0.418958	−12.063234	2.919267
**O**	0.034492	−13.902454	3.442467
**Ti**	−0.264748	−14.540617	5.074187
**O**	−1.221370	−13.372824	6.108382
**Ti**	−0.946457	−11.484075	6.866576
**O**	0.722267	−11.926226	7.627468
**Ti**	1.713322	−12.464792	6.172057
**O**	0.516610	−11.677156	5.050399
**P**	0.526600	−4.596975	1.751272
**O**	−0.480945	−3.546975	1.330969
**P**	−1.444003	−6.872133	2.356325
**O**	−1.943910	−8.242610	1.655627
**O**	1.820801	−4.363616	0.770422

**O**	0.984288	−4.683547	3.189146
**O**	−0.802384	−7.252996	3.717008
**O**	−2.580072	−5.884657	2.421572
**Ti**	−3.866488	−11.720768	7.216834
**O**	−2.796835	−11.320529	5.777001
**Ti**	−3.880533	−10.035911	4.943601
**O**	−4.062510	−10.729708	3.179660
**Ti**	−3.316030	−12.281535	2.596647
**O**	−2.086078	−12.458372	3.881540
**Ti**	−3.010547	−13.691270	5.070749
**O**	−2.179625	−15.245949	4.830460
**O**	−4.188039	−13.586594	3.534915
**O**	−4.114810	−13.420243	6.608156
**O**	−0.780950	−9.715032	6.514883
**O**	−2.234229	−11.636712	8.184448
**O**	−4.963447	−10.415748	6.421484
**O**	−2.828200	−8.636780	5.076926
**O**	1.252681	−11.375126	2.566214
**O**	2.998641	−11.366907	5.335549
**O**	1.413641	−14.272074	5.875667
**O**	−1.557669	−12.134290	1.538334
**H**	1.499926	−6.847998	−0.019844
**H**	0.377877	−8.143786	0.387090
**H**	2.393503	−6.894251	2.343863
**H**	1.176339	−8.085163	2.766376
**H**	2.167931	−9.642963	1.019883
**H**	3.504109	−8.494168	0.846070
**H**	−1.340633	−5.127213	0.061181
**H**	3.866583	−8.747789	3.242211
**H**	4.032879	−10.158984	2.439715
**H**	−1.653104	−9.039250	2.169987
**H**	2.611176	−4.828297	1.085389

$(TiO_2)_{10}$—alendronate (2)

**C**	−0.595322	−8.115197	9.756355
**O**	−1.224092	−9.245011	9.149281
**C**	−1.711844	−7.483047	10.596853
**C**	−1.405020	−6.261371	11.448662
**C**	−2.669026	−5.746086	12.108983
**N**	−2.376202	−4.611414	12.985622
**P**	0.840754	−8.739065	10.774030
**O**	1.655706	−9.669842	9.899222
**P**	−0.021312	−6.956916	8.413138
**O**	−1.377315	−6.426570	7.735391
**O**	0.071395	−9.620467	11.930101
**O**	1.538010	−7.620342	11.514294
**O**	0.778193	−5.801800	8.946914
**O**	0.716462	−7.812768	7.340068
**Ti**	0.375249	−9.255684	6.037605
**O**	2.119148	−9.467234	5.455164
**Ti**	2.348116	−10.941256	4.349560
**O**	0.889421	−11.929363	4.888413
**Ti**	−0.261264	−10.931590	2.930720
**O**	−0.298677	−12.781322	2.632061
**Ti**	−0.751513	−14.149351	3.729480
**O**	−1.213218	−13.695656	5.335436
**Ti**	−0.607317	−12.398665	6.754625
**O**	0.954507	−13.458364	6.995759
**Ti**	1.818380	−13.498197	5.360478
**O**	3.302219	−12.448434	4.797938
**Ti**	−3.443836	−11.784568	6.601319
**O**	−1.904001	−11.262640	5.839791
**Ti**	−2.499041	−9.565048	5.052519
**O**	−3.345054	−9.001245	3.391814
**Ti**	−3.062872	−10.315740	2.194189
**O**	−2.322389	−11.334200	3.553960
**Ti**	−3.509720	−12.798200	3.493947
**O**	−2.586202	−14.265472	3.020649
**O**	−4.252148	−11.796664	2.125950
**O**	−4.163671	−12.803691	5.247205
**O**	0.184016	−10.595980	7.169150
**O**	−1.992055	−12.666198	7.822254
**O**	−4.023257	−10.138341	6.167602
**O**	−1.804519	−8.325326	6.036640
**O**	−0.498673	−9.631946	4.349471
**O**	1.562458	−10.652599	2.690657
**O**	1.094559	−14.678231	4.216605
**O**	−1.280240	−10.205230	1.574938
**H**	−2.098202	−8.284756	11.242968
**H**	−2.526603	−7.239419	9.898592
**H**	−0.668863	−6.512120	12.226890
**H**	−0.961347	−5.458332	10.843402
**H**	−3.374965	−5.412911	11.336329
**H**	−3.161382	−6.579853	12.640229
**H**	−0.614485	−9.718966	8.539929

**H**	−1.782181	−4.934647	13.746992
**H**	−3.237770	−4.290641	13.420286
**H**	−1.688419	−7.098603	7.054610
**H**	−0.258689	−10.463144	11.583507

References

1. Frisch, M.J.; Trucks, G.W.; Schlegel, H.B.; Scuseria, G.E.; Robb, M.A.; Cheeseman, J.R.; Scalmani, G.; Barone, V.; Mennucci, B.; Petersson, G.A.; et al. *Gaussian 09, Revision, D.01*; Gaussian, Inc.: Wallingford, CT, USA, 2013.
2. Allard, M.M.; Merlos, S.N.; Springer, B.N.; Cooper, J.; Zhang, G.; Boskovic, D.S.; Kwon, S.R.; Nick, K.E.; Perry, C.C. Role of TiO_2 Anatase Surface Morphology on Organophosphorus Interfacial Chemistry. *J. Phys. Chem. C* **2018**, *122*, 29237–29248, doi:10.1021/acs.jpcc.8b08641.
3. Qu, Z.; Kroes, G.-J. Theoretical Study of Stable, Defect-Free $(TiO_2)n$ Nanoparticles with n = 10−16. *J. Phys. Chem. C.* **2007**, *111*, 16808–16817, doi:10.1021/jp073988t.
4. Zhao, Y.; Truhlar, D.G. The M06 suite of density functionals for main group thermochemistry, thermochemical kinetics, noncovalent interactions, excited states, and transition elements: Two new functionals and systematic testing of four M06-class functionals and 12 other functionals. *Theor. Chem. Account.* **2008**, *120*, 215–241, doi:10.1007/s00214-007-0310-x.
5. Zhao, Y.; Truhlar, D.G. Density Functionals with Broad Applicability in Chemistry. *Acc. Chem. Res.* **2008**, *41*, 157–167, doi:10.1021/ar700111a.
6. Zhao, Y.; Truhlar, D.G. Density Functional Theory for Reaction Energies: Test of Meta and Hybrid Meta Functionals, Range-Separated Functionals, and Other High-Performance Functionals. *J. Chem. Theory Comput.* **2011**, *7*, 669–676, doi:10.1021/ct1006604.
7. Wadt, W.R.; Hay, P.J. Ab initio effective core potentials for molecular calculations. Potentials for main group elements Na to Bi. *J. Chem. Phys.* **1985**, *82*, 284–298, doi:10.1063/1.448800.
8. Marenich, A.V.; Cramer, C.J.; Truhlar, D.G. Universal solvation model based on solute electron density and on a continuum model of the solvent defined by the bulk dielectric constant and atomic surface tensions. *J. Phys. Chem B.* **2009**, *113*, 6378–6396, doi:10.1021/jp810292n.
9. National Center for Biotechnology Information. PubChem Database. Alendronic acid, CID=2088. Available online: https://pubchem.ncbi.nlm.nih.gov/compound/Alendronic-acid (accessed 9 May 2020).
10. Bader, R.R.W. *Atoms in Molecules: A Quantum Theory*; Oxford University Press: Oxford, NY, USA, 1994.
11. Keith, T.A. *AIMAll (Version 17.01.25)*; TK Gristmill Software: Overland Park, KS, USA, 2017; Available online: aim.tkgristmill.com.
12. Bader, R.F.W. A Bond Path: A Universal Indicator of Bonded Interactions. *J. Phys. Chem. A.* **1998**, *102*, 7314–7323, doi:10.1021/jp981794v.
13. Bader, R.F.W.; Essén, H. The characterization of atomic interactions. *J. Chem. Phys.* **1984**, *80*, 1943–1960, doi:10.1063/1.446956.
14. Cremer, D.; Kraka, E. A Description of the Chemical Bond in Terms of Local Properties of Electron Density and Energy. *Croat. Chem. Acta* **1984**, *57*, 1259–1281.
15. Espinosa, E.; Molins, E.; Lecomte, C. Hydrogen bond strengths revealed by topological analyses of experimentally observed electron densities. *Chem. Phys. Lett.* **1998**, *285*, 170–173, doi:10.1016/S0009-2614(98)00036-0.
16. Espinosa, E.; Alkorta, I.; Rozas, I.; Elguero, J.; Molins, E. About the evaluation of the local kinetic, potential and total energy densities in closed-shell interactions. *Chem. Phys. Lett.* **2001**, *336*, 457–461, doi:10.1016/S0009-2614(01)00178-6.
17. Borissova, A.O.; Antipin, M.Y.; Karapetyan, H.A.; Petrosyan, A.M.; Lyssenko, K.A. Cooperativity effects of H-bonding and charge transfer in an L-nitroarginine crystal with Z′ > 1. *Mendeleev Commun.* **2010**, *20*, 260–262, doi:10.1016/j.mencom.2010.09.006.
18. Baryshnikov, G.V.; Minaev, B.F.; Minaeva, V.A.; Nenajdenko, V.G. Single crystal architecture and absorption spectra of octathio[8]circulene and sym-tetraselenatetrathio[8]circulene: QTAIM and TD-DFT approach. *J. Mol. Model.* **2013**, *19*, 4511–4519, doi:10.1007/s00894-013-1962-1.
19. Baryshnikov, G.V.; Minaev, B.F.; Korop, A.A.; Minaeva, V.A.; Gusev, A.N. Structure of zinc complexes with 3-(pyridin-2-yl)-5-(arylideneiminophenyl)-1HH-1,2,4-triazoles in different tautomeric forms: DFT and QTAIM study. *Russ. J. Inorg. Chem.* **2013**, *58*, 928–934, doi:10.1134/S0036023613080032.

20. Shahangi, F.; Chermahini, A.N.; Farrokhpour, H.; Teimouri, A. Selective complexation of alkaline earth metal ions with nanotubular cyclopeptides: DFT theoretical study. *RSC Adv.* **2014**, *5*, 2305–2317, doi:10.1039/C4RA08302D.
21. Puntus, L.N.; Lyssenko, K.A.; Antipin, M.Y.; Bünzli, J.C.G. Role of Inner-and Outer-Sphere Bonding in the Sensitization of EuIII-Luminescence Deciphered by Combined Analysis of Experimental Electron Density Distribution Function and Photophysical Data. *Inorg. Chem.* **2008**, *47*, 11095–11107, doi:10.1021/ic801402u.
